# Filovirus RNA in Fruit Bats, China

**DOI:** 10.3201/eid2109.150260

**Published:** 2015-09

**Authors:** Biao He, Yun Feng, Hailin Zhang, Lin Xu, Weihong Yang, Yuzhen Zhang, Xingyu Li, Changchun Tu

**Affiliations:** Academy of Military Medical Sciences, Jilin, China (B. He, L. Xu, X. Li, C. Tu);; Yunnan Institute of Endemic Diseases Control and Prevention, Dali, China (Y. Feng, H. Zhang, W. Yang, Y. Zhang);; Jiangsu Co-innovation Center for Prevention and Control of Important Animal Infectious Diseases and Zoonoses, Yangzhou University, Yangzhou, China (C. Tu)

**Keywords:** Bat, filovirus, zoonosis, genetic diversity, China, viruses, fruit bats, Rousettus leschenaultia

**To the Editor:** Filovirus-associated diseases, particularly those caused by Ebola and Marburg viruses, represent major threats to human health worldwide because they have extremely high death rates and antiviral therapies or vaccines against them are not available (*1*). Members of the family *Filoviridae* are classified into 3 genera: *Marburgvirus*, *Ebolavirus*, and the recently approved *Cuevavirus* (*2*,*3*). Marburg virus (MARV) and Ebola virus (EBOV) were initially isolated in Africa, but other filoviruses have been identified on other continents. The initial *Cuevavirus*, Lloviu virus (LLOV), was identified in Europe (Spain) (*3*), and Ebola-Reston virus has been found in pigs in Asia (the Philippines) (*4*). 

Bats are natural reservoirs for filoviruses (*5*). Viral isolation and serologic studies indicate that filovirus infections have occurred in various bat species in central Africa countries (*6*), the Philippines (*7*), China (*8*), and Bangladesh (*9*). However, identification of these viruses in bats has been difficult; although isolates of MARV have been obtained (*6*) and the genome of LLOV has been fully sequenced (*3*), very short sequences of EBOV have been obtained from bats, and only in Africa (*5*). Reports of molecular detection or isolation of filoviruses in bats in Asia are lacking. We conducted a study to investigate the presence of filoviruses in bats in China.

In June 2013, twenty-nine apparently healthy *Rousettus leschenaultia* fruit bats were captured in Yunnan Province, China. All bats were humanely killed, and their intestines, lungs, livers, and brains were collected and subjected to viral metagenomic analysis by a previously described method (*10*). As a result, we obtained and reassembled de novo 10 million reads into 590,010 contigs. Of these contigs, 3 (129–354 nt) were genetically close to filovirus, corresponding to the nucleoprotein gene of LLOV (74% nt identity), the viral protein 35 gene of Sudan Ebola virus (69% nt identity), and the L gene of Tai Forest Ebola virus (72% nt identity) (Technical Appendix Table 1).

For further screening, we used the longest contig as a template for design of specific seminested primers. Nested degenerate primer pairs were also designed and focused on the most conserved region of the L gene of all currently known filoviruses (Technical Appendix Table 2). After screening, 2 reverse transcription PCRs of tissues from 1 bat (Bt-DH04) showed positive amplification in specimens from its lung but not from intestine, liver, or brain tissue. Moreover, 5 blind passages in Vero-E6 cells failed to isolate the virus from the lung homogenate. In an attempt to obtain its genomic sequence, 24 primer pairs covering the full genome were further designed by alignment of these contig sequences with the full genomes of representative filoviruses within the 3 genera. All amplifications used ddH_2_O as a negative control; positive controls were not available because filoviruses were not available in China. Two fragments of 2,750-nt (F1) and 2,682-nt (F2) were successfully amplified from lung tissue of Bt-DH04; attempts to amplify the remaining regions failed. Alignment with sequences of 26 representative filoviruses of 7 species from 3 genera revealed that F1 covered the 3′ end of the nucleoprotein gene and almost the entire viral protein 35 gene, and that F2 covered the middle region of the L gene, corresponding to nt 1,313–4,085 and nt 12,613–15,302 of the full genome of EBOV (GenBank accession no. HQ613402). The 2 fragment sequences were submitted to Genbank (accession no. KP233864), and the strain has been tentatively named Bt-DH04. 

Phylogenetic analysis showed that the Bt-DH04 strain is placed, together with LLOV, at basal position and intermediate between EBOV and MARV (Figure). It is divergent from all known filoviruses, with F1 sharing the highest nucleotide identities (46%–49%) to members of the genus *Ebolavirus*, followed by 44% to LLOV and <40% to MARV (Figure, panel A). The L gene is the most conserved region of filoviruses, and F2 of Bt-DH04 strain shared relatively closer 66%–68% nt identities with members of the genus *Ebolavirus*, followed by 64% with LLOV and ≈60% with MARV (Figure, panel B). This sequence diversity is likely the main factor for unsuccessful amplification of the full genome of Bt-DH04.

**Figure:**
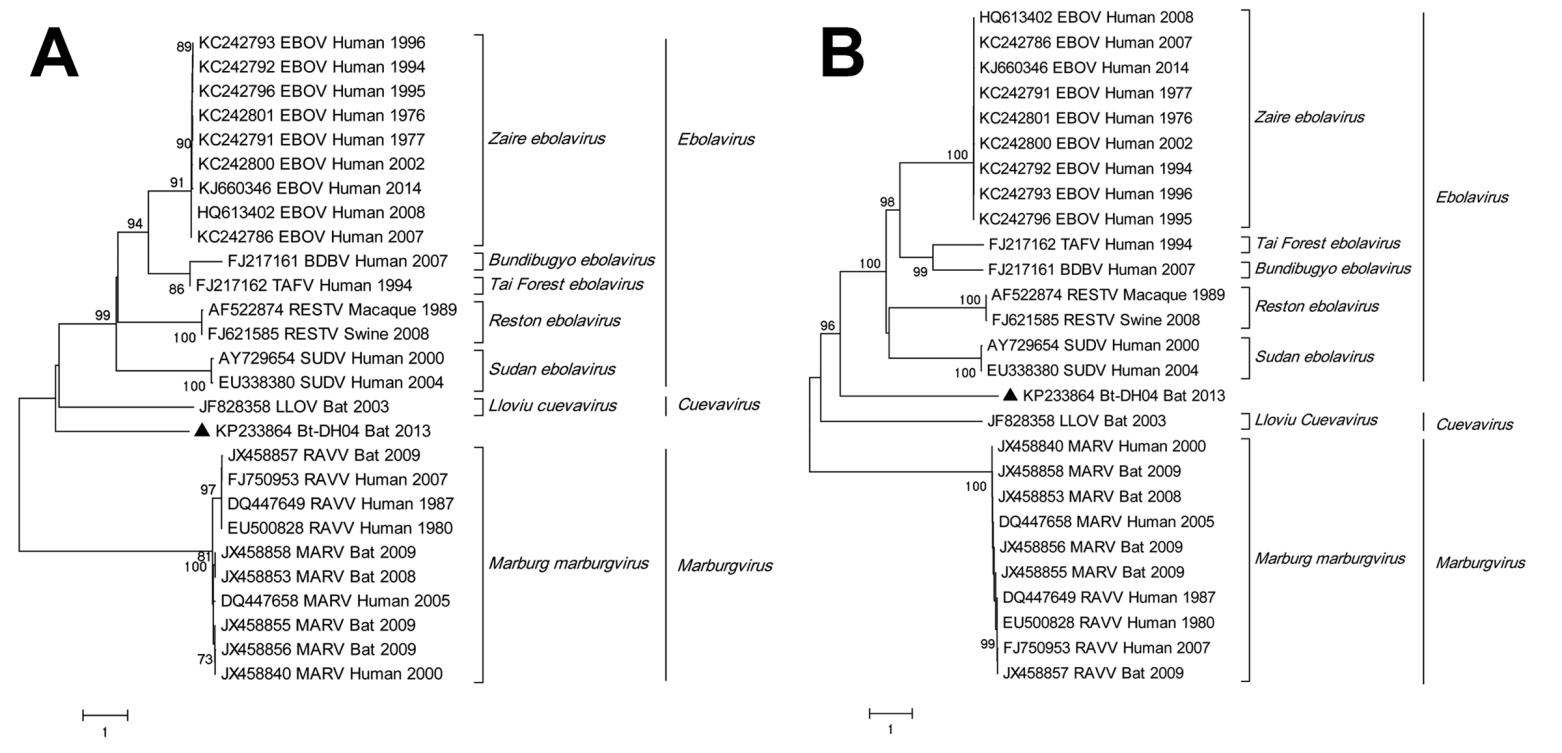
Phylogenetic analysis of 2 fragments of filovirus Bt-DH04 and other filoviruses. Full genomes of representatives from the family *Filoviridae* were trimmed and aligned with F1 (partial nucleoprotein/viral protein 35 gene, panel A) and F2 (middle L gene, panel B) of filovirus strain Bt-DH04 by using ClustalW version 2.0 (http://www.clustal.org), then phylogenetically analyzed by using MEGA6 (http://www.megasoftware.net) by the maximum-likelihood method, resulting in a bootstrap testing value of 1,000. Sequences are listed by their GenBank accession numbers, followed by the virus name, host, and collection time. Triangles identify the novel filovirus strain Bt-DH04 (China). Scale bars indicate nucleotide substitutions per site.

Increasing PCR evidence has identified the existence of filoviruses in bats in Africa and Europe (*3*,*5*); however, although serologic studies have shown that filovirus antibodies are prevalent in bats in a few countries in Asia (e.g., the Philippines, Bangladesh and China [*7*–*9*]), filovirus or filovirus RNA have not been reported in bats in Asia. Our results show that the Bt-DH04 strain is likely a novel bat-borne filovirus in Asia and provide evidence that bats in Asia harbor more divergent filoviruses than previously thought.

Fruit bats in the genus *Rousettus* are widely distributed throughout Southeast Asia, South China, and the entire Indian subcontinent and have had positive serologic results for Ebola viruses in these regions (*7*–*9*), indicating that these bats play a role in the circulation of filoviruses in Asia. The possibility of new emerging filovirus-associated diseases in the continent emphasizes the need for further investigation of these animals.

Technical Appendix Sample collection, preparation, and outcomes of testing for filoviral RNA in fruit bats, China.
