# Supplementary material for: Filovirus RNA in Fruit Bats, China
Source: Emerg Infect Dis. 2015 Sep;21(9):1675–7. doi: 10.3201/eid2109.150260 (PMC4550138; doi:10.3201/eid2109.150260)
Supplement: Technical Appendix — Sample collection, preparation, and outcomes of testing for filoviral RNA in fruit bats, China. [file 15-0260-Techapp-s1.pdf]

Article DOI: http://dx.doi.org/10.3201/eid2109.150260

Filovirus RNA in Fruit Bats, China

Technical Appendix

Sample collection, preparation, and outcomes of testing for filoviral RNA in fruit bats, China

In June 2013, 29 apparently healthy *Rousettus leschenaulti* bats were captured by using a bird net in a longan orchard in Yunnan province of China. Living bats were euthanized by intravenous injection of potassium chloride at the local Center for Animal Diseases Control and Prevention. We intended to focus on encephalitis-related viruses in brains, respiratory tract-borne viruses in lungs, and viruses in digestive system, therefore the brains, lungs, livers and intestines of each bat were collected separately into cryotubes and immediately frozen in a liquid nitrogen tank prior to transportation to the laboratory, where they were stored at –80°C.

Bat samples were prepared for metagenomic analysis as described by He et al. (*1*). The intestines, lungs, livers, and brains of 29 bats were pooled together and homogenized in SM buffer (1:10 [w/v]; 50 mM Tris, 10 mM MgSO4, 0.1 M NaCl, pH7.5). The homogenized samples were centrifuged at 8,000×g at 4□C for 30 min to remove cell debris and foreign materials, and the supernatants were immediately filtered through 0.45-μm and 0.22-μm filters (Millipore). Host genomes and other free nucleic acids were eliminated by digestion of nuclease mixture containing DNase (Ambion), Benzonase Nuclease (Novagen) and RNase I (Fermentas).

The viral RNAs were then extracted immediately using QIAamp® Viral RNA Mini Kit (Qiagen) according to the manufacturer's protocol. Total viral RNAs were dissolved in RNase-free H2O and used immediately for the following reverse transcription with SuperScript III reverse transcriptase (Invitrogen) using anchored random primers according to the manufacturer's protocol. To synthesize dscDNA, a Klenow fragment (New England Biolabs)

was added to the cDNA mixture, and incubated at 37°C for 60 min. After inactivation of the enzyme, phosphates and free single-stranded bases in the dscDNA reaction was removed using shrimp alkaline phosphatase and exonuclease I (TaKaRa).

To obtain sufficient viral nucleic acid, single primer amplification was employed to amplify the dscDNA with the Accuprime Taq DNA Polymerase System (Invitrogen) according to the manufacturer’s protocol. Briefly, a 50 μl reaction system containing 10 μl of the above dscDNA mixture, random primers (20 mM), 10×Accuprime buffer I, and Taq DNA Polymerase (1 U) was denatured at 94°C for 2 min, followed by 40 cycles of 94°C denaturing for 30 s, 54°C annealing for 30 s, 68°C extending for 1 min with final 68°C extension for 8 min. The PCR products were then purified using the QIAquick PCR Purification Kit (Qiagen) and dissolved in 50 μl TE buffer (100 mM Tris-HCl, 10 mM EDTA, pH8.0). The purified PCR products were pooled together and then subjected to HiSeq 2000 (Solexa) sequencing in 1 lane by the Beijing Genome Institute (BGI).

To screening filovirus in these samples, the longest contig generated by viral metagenomic analysis was used as a template for design of specific semi-nested primers (Filo-F/Filo-in-F/Filo-R). Nested degenerate primer pairs (FV-F1/FV-R1, FV-F2/FV-R2) were also designed targeting the most conserved region of the L gene of all currently known filoviruses. Viral RNA of each tissue was extracted as above description; cDNA was synthesized by random hexamers and SuperScript III reverse transcriptase (Invitrogen). The 2 PCR methods were conducted by the same Master PCR Mix (Tiangen) and program: denaturing at 94°C for 2 min, followed by 30–35 cycles of 94°C denaturing for 30 s, 54□C annealing for 30 s, 72°C extending for 40 s with final 72°C extension for 5 min. PCR products with expected size were directly sequenced by an ABI 3730 DNA Analyzer (Invitrogen).

The positive sample obtained by above RT-PCR screening was used for full genome amplification. A total of 2 dozen degenerate primer pairs covering the full genome were further

designed by alignment of these contigs with the full genomes of representative filoviruses within the 3 genera. The positive cDNA and LA Taq DNA polymerase (TaKaRa) were used to amplify full genome according to the manufacturer’s protocol. The reaction system was denatured at 94°C for 2 min, followed by 40 cycles of 94°C denaturing for 30 s, 54-57°C annealing for 30 s, 72°C extending for 1–3 min with final 72 □C extension for 7 min. Negative control was ddH2O, but positive control was not considered due to filoviruses not available in China as well as a lack of corresponding biosafety facility. Positive amplicons were purified and ligated into the pMD18-T vector (TaKaRa), used to transfect TOP10 chemically competent Escherichia coli (Tiangen), and then sequenced by an ABI 3730 DNA Analyzer (Invitrogen). Of each amplicon, 5 clones were sequenced.

Technical Appendix Table 1. Sequences of contigs generated by viral metagenomic analysis and their identities to filoviruses*

Contig	bp	Sequence (5′→3′)	Gene	Nucleotide identity	Virus (Genbank Accession no./location)
1	207	AATAATCTGGAACATGGATTGTATCCTCAGTTATCAGCTAT<br>AGCAATAGGTATTGCAACTGCACATGGCAGCACACTTGGA<br>GGTGTTAATGTAGGTGAACAATTTCAACCATTGCGTGAAG<br>CTGCAACAGAGGCAGAAAAGCAGCTACAACGGTACACAG<br>AAATAAAGGAATTGGACCAGCAAGGTTTAGATGATCAAGA<br>ACGTAAG	NP	74%	Lloviu virus (JF828359/1,395-1,601)
2	354	GTGAAATGCTGTCCCGAACAATTGAAAGATGCTCATGAGA<br>ATTTGACTCGTGTTAATTCATTAACTGAAAAAACTTTTGCTA<br>AACCTAGTTTTACAGCTAAAGAACTTAGAGATATGATATAT<br>GATCATCTTCCAGGATATGAGACAGCATTTCACCAATTAAC<br>ACAGGTAATATGCAAGATTGCAAAAGATGAGGGTCAATTG<br>GAGCAAGTTCATACAGAATTTCAATCCTCCTTAGCTGAAG<br>GTGATTCTCCACAAAGTGCATTAATACAATTGACTAAACGC<br>ATGACTATTTTCGACGGAAGATCACCTCCACTGATTCATAT<br>AAACACACGAGCAGCCTACGACGACGAT	VP35	69%	Sudan ebolavirus (KC24278/3,668-4,022)
3	129	TTCTCTGGATTCAAAAAGGACACAGACATTGGGCTATTAG<br>CACTCAAAAAGCCATTAGATTATAGTACAATTGTTGTCACC<br>TTATCAATACCCCAAGTCTTAGGGGGATTATCATTTTTGAA<br>TCCAGAG	L	72%	Tai Forest ebolavirus (FJ217162/14,191-14,319)

Contig	bp	Sequence (5′→3′)	Gene	Nucleotide identity	Virus (Genbank Accession no./location)

*NP, nucleoprotein; VP, viral protein.

Technical Appendix Table 2. Primers used in this study. Primers Filo-F, Filo-in-F, Filo-R, FV-F1, FV-R1, FV-F2 and FV-R2 are used to screen filovirus in samples, the rest are used to amplify full genome. The locations of primers refer to the position on the genome of EBOV (HQ613402).

Primer	Sequence (5′→3′)	Polarity	Location
Filo-F	TGATATATGATCATCTTCCAGG	+	3,727-3,748
Filo-in-F	GCATTTCACCAATTAACACAGG	+	3,759-3,780
Filo-R	TTTATATGAATCAGTGGAGGTG	–	3,920-3,941
FV-F1	GCMTTYCCIAGYAAYATGATGG	+	13,261-13,282
FV-R1	GTDATRCAYTGRTTRTCHCCCAT	–	13,713-13,739
FV-F2	TDCAYCARGCITCDTGGCAYC	+	13,316-13,336
FV-R2	GIGCACADGADATRCWIGTCC	–	13,646-13,666
F1	ATCTGGAACATGGMYTGTATCC	+	1,308-1,329
F2	GGAACATGGMYTGTATCCTCAG	+	1,312-1,333
R1	GYTTYTCWGCCTCTGTTGCAGC	–	1,424-1,445
F3	ACTGCTGCAGCTACTGAAGCTTACTGG	+	3,510-3,536
F4	AAAGATGCTCATGAGAATTTGACTCG	+	3,780-3,805
F5	TCCTTAGCTGAAGGTGATTCTCC	+	3,843-3,865
F6	CCAGGATATGGGACAGCATTTCACC	+	3,889-3,913
F7	AAGATTGCAAAAGATGAGGGTCAATTGG	+	3,933-3,960
F8	CCTCCTTAGCTGAAGGTGATTCTCC	+	3,986-4,010
F9	CARAAVWYVTAYAGYTTTGAYTCAAC	+	4,704-4,729
R2	GCDGMNGYNGTTGARTCAAARC	–	4,717-4,738
R3	GGNACACCDGHHCKRAADSCCCA	–	6,213-6,235
F10	YCCHMGNTGYCGBTATGTICAC	+	6,353-6,374
R4	TGTGNACATAVCGRCANCKDGG	–	6,354-6,375
F11	CRGACACACAAAAASAADRRA	+	7,237-7,257
F12	TGGACDGGNTGGMRRCARTGG	+	7,889-7,909
R5	AYCCAYTGYYKCCANCCHGTCC	–	7,890-7,911
F13	VVDTTYGARGCHGCVYTRTGGC	+	9,088-9,109
F14	GCHGCVYTRTGGCARSRDTGGG	+	9,097-9,118
F15	WGCVYTRTGGCARCADTGGG	+	9,099-9,118
R6	GAYYSYCKRTCCCAHTGYTGCC	–	9,107-9,128
R7	GTCAKBGHCCAKGCWGGDGC	–	10,459-10,478
F16	GARTAACTAYGARGAAKATTAA	+	11,410-11,431

Primer	Sequence (5′→3′)	Polarity	Location
F17	CCHATHGTNYTVGAYCARTGTG	+	11,545-11,566
F18	CCHATWRTBYTVGAYCARTGTGA	+	11,545-11,567
F19	TNCARAARCAYTGGGGBCAYCC	+	12,611-12,632
R8	TAGTTCATTGTGGAGTACAGGATGCC	–	12,627-12,652
R9	GTAATTTCAACTTTGTGGCATG	–	12,673-12,694
R10	CACTGGTACTAGTTCTATTGTGATGCC	–	13,331-13,357
R11	CTAGTGAACTCATATCTAAAGGC	–	13,414-13,436
R12	AATGGTCTAGTGAACTCATATCTAAAGGC	–	13,414-13,442
R13	GGTCTAGTGAACTCATATCTAAAGGC	–	13,415-13,440
R14	TTTGGCTTGTTAGACAGTGAGGTGG	–	13,543-13,567
R15	AATTTGAGCACATGATATGCTAGTCC	–	13,647-13,672
F20	GATTAACATGGGCAACGCAAGG	+	15,173-15,194
F21	GTGTTAATTTGGAGGTTGAGG	+	15,240-15,260
F22	TGTTACCAGTACATTATTCAGG	+	15,272-15,293
R16	GWRTTRCTCATNCKRTTBGCCAT	–	15,340-15,362
F23	GCNAGYAAYTTYTTYCAYGCDTC	+	16,393-16,415
R17	TCRTMHARYTTRTAATGCATKGA	–	16,933-16,955
R18	CYTCRTCNARYTTRTARTGCATKG	–	16,934-16,957
R19	GCHARKGAHGCRTGRAARAARTTRC	–	16,937-16,421
R20	TYYTYDGTNGTYTCWGCATCCAT	–	17,275-17,297
R21	WRTTYYCWRTHGTYTCWGCATCC	–	17,277-17,299

Reference

<jrn>1. He B, Li Z, Yang F, Zheng J, Feng Y, Guo H, et al. Virome profiling of bats from Myanmar by metagenomic analysis of tissue samples reveals more novel mammalian viruses. PLoS ONE. 2013;8:e61950. http://dx.doi.org/10.1371/journal.pone.0061950</jrn>
